# The presence of β’1-COP and β’2-COP is required for female and male gametophyte development

**DOI:** 10.1007/s00497-023-00467-6

**Published:** 2023-06-02

**Authors:** Judit Sánchez-Simarro, Fernando Aniento, María Jesús Marcote

**Affiliations:** Departamento de Bioquímica y Biología Molecular, Facultad de Farmacia, Instituto Universitario de Biotecnología y Biomedicina (BIOTECMED), Universitat de València, 46100 Burjassot, Spain

**Keywords:** Arabidopsis, Plant development, Gametophyte, Coat protein I (COPI), β’-COP

## Abstract

Coat protein I (COPI) and Coat protein II (COPII) coated vesicles mediate protein transport in the early secretory pathway. Although several components of COPII vesicles have been shown to have an essential role in Arabidopsis gametogenesis, the function of COPI components in gametogenesis has not been studied in detail. COPI consists of a heptameric complex made of α, β, β′, γ, δ, ɛ, and ζ-COP subunits and most subunits have several isoforms in Arabidopsis. We have found that two isoforms of the β’-COP subunit, β’1-COP and β’2-COP, are required for female and male gametophyte development. Reciprocal crosses between wild type plants and plants heterozygous for T-DNA insertions in *β’1-COP* and *β’2-COP* showed that* β’1β’2-cop* gametophytes are not transmitted.

## Introduction

The biosynthetic secretory pathway transports newly synthesized proteins and lipids from the endoplasmic reticulum (ER) to the plasma membrane and/or the extracellular space. Transport along the early secretory pathway (ER-Golgi) is mediated by vesicles coated by Coat Protein complexes (COPs). Anterograde (ER to Golgi) transport is mediated by COPII vesicles while retrograde (Golgi to ER) and/or intra-Golgi transport (either in the *cis*–*trans* and/or *trans*/*cis* direction) is mediated by COPI vesicles (Aniento et al. 2022; Pereira and Di Sansebastiano 2021). The COPII complex consists of five proteins that have different paralogs: the GTPase secretion-associated Ras-related protein Sar1A-E, SEC23A-G, SEC24A-C, SEC13A-B and SEC31A-B (Chung et al. 2016). The main component of the COPI coat is the coatomer complex, which is composed of seven subunits (α/β/β'/γ/δ/ε/ζ), which interact with Golgi membranes via the GTPase ADP-ribosylation factor 1 (ARF1) (Aniento et al. 2022). The coatomer complex is not only involved in the biogenesis of COPI vesicles but it is also required to select the cargo to be included in the vesicles. Conceptually (and biochemically), subunits of the coatomer complex can be grouped into two subcomplexes, an outer (α/β′/ε) subcomplex and an inner (β/δ/γ/ζ) subcomplex, although subunits from both subcomplexes appear to be involved in cargo recognition (including α-, β´-, γ- and δ-COP) (Aniento et al. 2022). In mammals, γ-COP and ζ-COP subunits have two isoforms, in contrast to yeast, that contains only one isoform for every subunit (Gao et al. 2014). Although COPI vesicles formed in vitro with different COPI subunit isoforms had the same protein composition (Adolf et al. 2019), it was postulated that different populations of COPI vesicles (perhaps with different cargo proteins) could be formed by different isoforms of the γ- and ζ-COP subunits (Popoff et al. 2011). Indeed, γ1-COP and not γ2-COP has been shown recently to specifically promote neurite outgrowth (Jain Goyal et al. 2020). The plant COPI complex has been found to play specific roles in growth and development, cell plate formation during cytokinesis and Golgi morphology (Ahn et al. 2015; Woo et al. 2015; Gimeno-Ferrer et al. 2017; Sánchez-Simarro et al. 2020). In Arabidopsi*s*, most COPI genes (including α-, β-, β’, ε- and ζ-COP) have 2–3 paralogs and it is not yet known whether different COPI subunit isoforms may have specific functions or else are functionally redundant (Gao et al. 2014). Interestingly, two morphologically different types of COPI vesicles have been identified in Arabidopsis (Donohoe et al. 2007), which might be formed by different COPI subunit isoforms, although this postulate needs to be confirmed.

Plant reproduction is one of the most fundamental plant processes. It is not only essential to perpetuate plant species and for plant evolution, but also important for crop production and agriculture economy. It has been reported that factors involved in vesicular trafficking play a key role in plant gametophyte development (El-Kasmi et al. 2011; Liu F et al. 2021; Rojek et al. 2021; Zhou et al. 2022). Several COPII components (SEC24A-C and SEC31A-B) have been shown to have an essential role in Arabidopsis gametogenesis. A *SEC24A* loss of function mutation caused defects in pollen leading to failure of male transmission of the *SEC24A* mutation (Conger et al. 2011) and a significant decrease of *SEC24B* and *SEC24C* expression affected male and female gametogenesis (Tanaka et al. 2013). Similarly, a *sec31Asec31B* double mutant was unavailable due to the lethality of male and female *sec31Asec31B* gametophytes (Liu X et al. 2021).

Concerning components of the COPI coat, very little is known about their role in reproduction. COPI-mediated membrane trafficking has been postulated to play a role in cytokinesis in Drosophila male meiotic divisions (Kitazawa et al. 2012) and mammalian spermatogenesis (Moreno et al. 2000). In plants, it has been shown that Arabidopsis COPI subunit isoforms are required for the acceptance of compatible pollen (Cabada Gomez et al. 2020). Individual anthers from flowers of Col-0 wild-type or knockout lines of isoforms of the α-COP, γ-COP, and ε-COP subunits were used to manually apply pollen grains to the stigmas of the emasculated flowers. Altered compatible pollen grain adherence and tube germination and reduced seed set was observed in *α1-cop*, whereas the other lines had milder phenotypes but visibly retarded compatible pollen acceptance (Cabada Gomez et al. 2020). However, no more data are available yet about the involvement of COPI function in plant reproductive processes. Recently, we have shown that the three genes (*β’1–3-COP*) that encode the three Arabidopsis β’-COP isoforms are at least partially redundant as none of the loss-of-function single mutants of these genes display severe developmental defects under standard growth conditions (Sánchez-Simarro et al. 2022). β’-COP double mutant analysis showed that *β’2-COP* (At3g15980) cannot compensate for the simultaneous loss of *β’1-COP* (At1g52360) and *β’3-COP* (At1g79990) since the *β’1β’3-cop* double mutant failed to develop beyond the seedling stage. However, *β’2β’3-cop* double mutants have no major phenotypic alterations under standard growth conditions, indicating that *β’1-COP* does seem to compensate for the simultaneous lack of* β’2-COP* and *β’3-COP*. Finally, *β’3-COP* cannot compensate for the simultaneous loss of* β’1-COP* and *β’2-COP* as no *β’1β’2-cop* double mutants could be obtained (Sánchez-Simarro et al. 2022). In this study we decided to explore whether the simultaneous loss of function of *β’1-COP* and *β’2-COP* was gametophytically lethal. We found that *β’1β’2-cop* female and male gametophytes are not transmitted to the progeny.

## Materials and methods

### Plant material

*Arabidopsis thaliana* (ecotype Col-0) was used as wild type. The loss-of-function mutants *β’1-cop-1* (SALK_206753) and *β’2-cop-1* (SALK_056771) were from the Salk Institute Genomic Analysis Laboratory and obtained from the Nottingham Arabidopsis Stock Centre. *A. thaliana* plants were grown in growth chambers under a 16-h-light:8-h-dark regime at 22 °C. The progeny of the different crosses was characterized by PCR (Sánchez-Simarro et al. 2022).

### Silique clearance

Mature siliques were cleared with 0.2N NaOH and 1% SDS solution. Measurements of the seeds and siliques were made using the ImageJ software.

### Statistical analysis

Differences in silique length, seed number and seed set, between wild type and *β’1*^+*/─*^* β’2*^*─/─*^, were tested using a two-sample t-test with unequal variances (Microsoft Excel 2013).

## Results and discussion

**Fig. 1:**
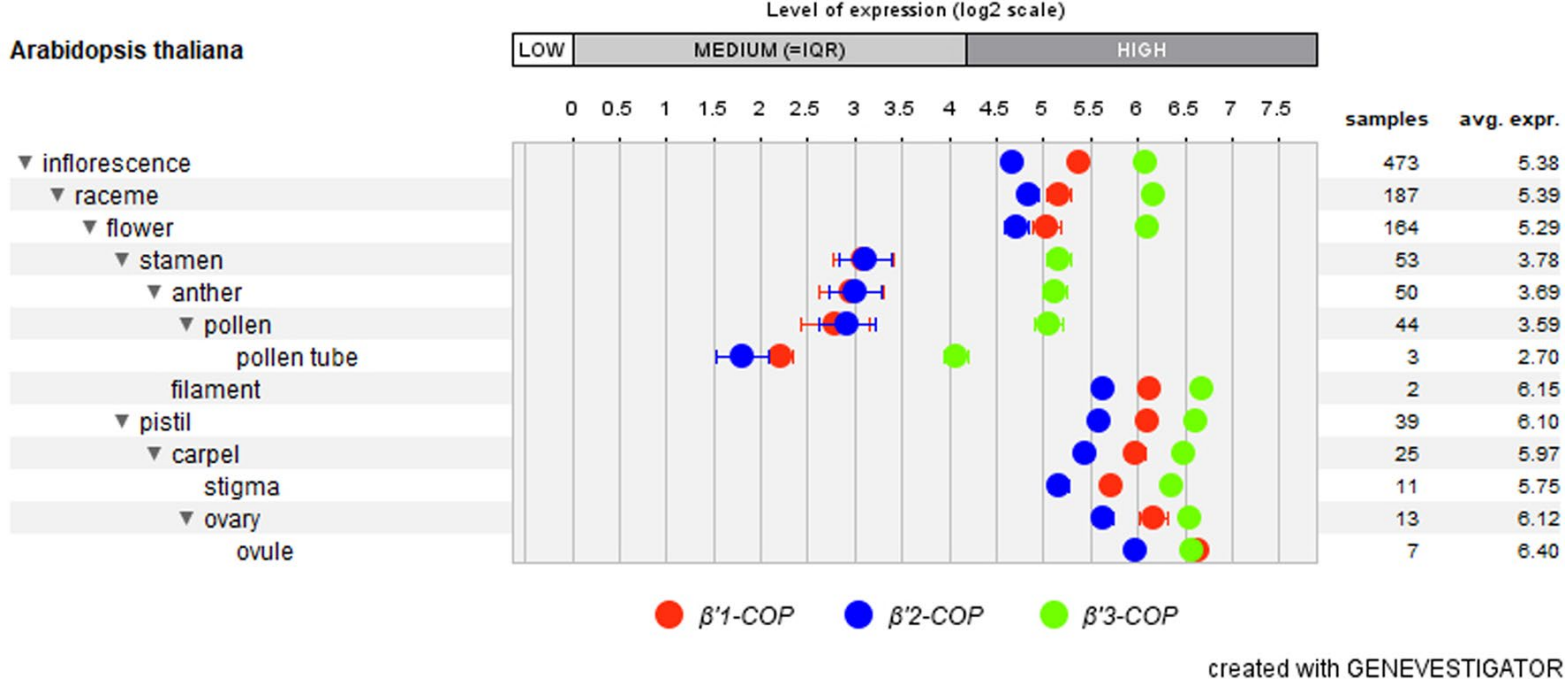
Expression patterns of *β’1-COP*, *β’2-COP* and *β’3-COP* in reproductive tissues. Data obtained by GENEVESTIGATOR. The number of samples aggregated in each category to calculate this average is indicated on the right. HIGH”, “MEDIUM” and “LOW” expressions are determined by looking at all expression values of all genes over all samples (www.genevestigator.com, accessed on 24 November 2022)

Arabidopsis *β’-COP* genes are widely expressed (Sánchez-Simarro et al. 2022). Data from microarray experiments obtained from public databases such as Genevestigator (www.genevestigator.com; Zimmermann et al. 2004) showed medium–high levels of expression of the three genes in female and male reproductive tissues (Fig. 1).

We have previously characterized single and double *β’-COP* mutants (Sánchez-Simarro et al. 2022). To obtain the double mutants, reciprocal crosses in both directions were performed with the single mutants (Sánchez-Simarro et al. 2022). Under standard growth conditions, *β’1β’3-cop* double mutants display developmental defects and *β’2β’3-cop* double mutants showed a wild type phenotype. However, no homozygous double mutants were obtained when *β’1-cop-1* and *β’2-cop-1* plants were crossed (Sánchez-Simarro et al. 2022). In this study, we analyzed the progeny segregation in detail. We screened 100 F2 plants and the results are summarized in Table 1. Not only *β’1*^*─/─*^* β’2 *^*─ /─*^ plants were not found but neither trans-heterozygous *β’1*^+*/─*^* β’2*^*─/─*^ and *β’1*^*─/─*^* β’2*^+*/─*^ plants could be identified, suggesting that *β’1β’2-cop* gametophytes may not be transmitted

**Table 1:** Segregation of the selfed progeny of *β’*1^+/─^* β*’2^+/─^

Parental genotype ♀ × ♂	Progeny genotype									F1 seedlings	*χ*^2^	
	*β’*1^+/+^ *β’*2^+/+^	*β’*1^+/+^ *β’*2^+/─^	*β’*1^+/+^ *β’*2^−/─^	*β’*1^+/─^ *β’*2^+/+^	*β’*1^+/─^ *β’*2^+/─^	*β’*1^+/─^ *β’2*^−/─^	*β’*1^−/─^ *β’2*^+/+^	*β’*1^−/─^ *β’2*^+/─^	*β’*1^−/─^ *β’2*^−/─^	(*n*)	Actual	Expected
*β’*1^+/−^ *β’*2^+/−^ × *β’*1^+/−^ *β’*2^+/−^	15 (6.25)	24 (12.5)	10 (6.25)	20 (12.5)	25 (25)	0 (12.5)	6 (6.25)	0 (12.5)	0 (6.25)	100	60.84	15.51

It is shown the expected segregation when gametogenesis is not affected in brackets. Results of the Chi-square (*X*^2^) distribution analysis are indicated (*P *= 0.05)

.

**Fig. 2:**
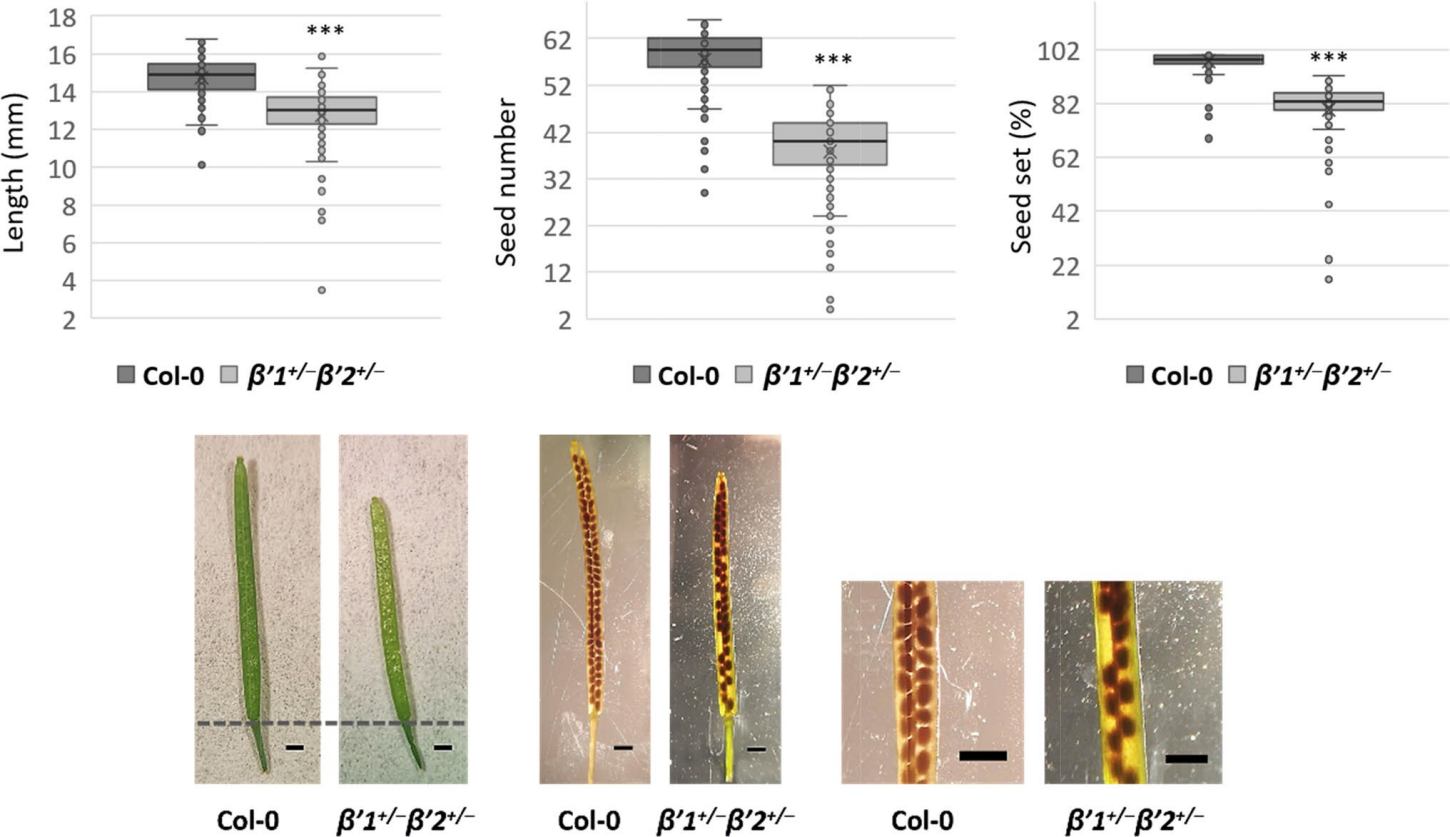
Silique length, seed set and seed number in *β’1*^+*/─*^* β’2*^+*/─*^. Top panel: statistical analysis of the silique length, seed setting rate and seed number in wild type (Col-0) and *β’1*^+*/─*^* β’2*^+*/─*^. The boxes display the interquartile range and the thick line shows the median. Whiskers of the box plots display the maximum and minimum values.* n* ≥ 100. Significance by Student's *t*-test: ***,* P*-value < 0.001. Bottom panel: representative photographs of siliques of the wild type (Col-0) and *β’1*^+*/─*^* β’2*^+*/─*^mutant. Scale bars = 1 mm

We observed that the siliques of *β’1*^+*/─*^* β’2*^+*/─*^ plants were shorter than wild type and they have a reduced seed number (Fig. 2). In addition, they had a reduced seed set when compared to wild type (Fig. 2). These results support our conclusion that the presence of both *β’1-COP* and *β’2-COP* is required for gametogenesis.

To investigate the gametophyte specificity for the defect of *β’1β’2-cop* co-transmission, reciprocal crosses of the double heterozygous *β’1*^+*/─*^* β’2*^+*/─*^ with wild type were performed. When *β’1*^+*/─*^* β’2*^+*/─*^ was used as the male parent and wild type as the female parent, no *β’1*^+*/─*^* β’2*^+*/─*^ plants were obtained from the F1 generation (Table 2). Similarly, when wild type was used as the male parent and *β’1*^+*/─*^* β’2*^+*/─*^ as the female parent, no *β’1*^+*/─*^* β’2*^+*/─*^ plants were found (Table 2). These results indicate that male and female *β’1β’2-cop* gametophytes could not be transmitted.

**Table 2:** Segregation data from the reciprocal crosses between *β’*1^+/─^* β*’2^+/─^ and wild type (*β'*1^+/+^* β*’2^+/+^). It is shown the expected segregation when gametogenesis is not affected in brackets

Parental genotype ♀ × ♂	Progeny genotype				F1 seedlings (*n*)	*χ*^2^	
	*β’*1^+/+^ *β’*2^+/+^	*β’*1^+/+^ *β’*2^+/─^	*β’*1^+/−^ *β’*2^+/+^	*β’*1^+/─^ *β’*2^+/−^		Actual	Expected
WT × *β’*1^+/−^*β’*2^+/−^	40 (21.5)	39 (21.5)	7 (21.5)	0 (21.5)	86	61.44	7.82
*β’*1^+/−^*β’*2^+/−^ × WT	31 (23)	24 (23)	37 (23)	0 (23)	92	34.34	7.82

Results of the Chi-square distribution (*X*^2^) analysis are indicated (*P* = 0.05)

Therefore, this work indicates that COPI vesicle trafficking is crucial for Arabidopsis gametogenesis. This work also reinforces the hypothesis that Arabidopsis COPI subunit isoforms may have both redundant and non-redundant functions. While β’1- and β’2-COP seem to have at least partially redundant functions in gametogenesis (each one can substitute the other one), β’3-COP cannot replace the absence of both β’1- and β’2-COP. In addition, previous studies showing altered pollen–pistil interaction phenotypes of *α1-cop* mutants might be explained by α1-COP playing a more predominant role than α2-COP in gametophyte development (Cabada Gomez et al. 2020). Future experiments should investigate whether different COPI subunit isoforms may be part of different vesicle populations (Donohoe et al. 2007) with specific functions in different physiological processes.

### Author’s contribution statements

JSS, FA and MJM conceived and designed research. JSS conducted experiments. MJM and FA wrote the manuscript. All authors read and approved the manuscript. The authors declare that their results have not been submitted for publication elsewhere. All co-authors agree to share equal responsibility for the content of the manuscript.

## References

[CR1] Adolf F, Rhiel M, Hessling B, Gao Q, Hellwig A, Béthune J, Wieland FT (2019). Proteomic profiling of mammalian COPII and COPI vesicles. Cell Rep.

[CR2] Ahn HK, Kang YW, Lim HM, Hwang I, Pai HS (2015). Physiological functions of the COPI complex in higher plants. Mol Cells.

[CR3] Aniento F, de Medina S, Hernández V, Dagdas Y, Rojas-Pierce M, Russinova E (2022). Molecular mechanisms of endomembrane trafficking in plants. Plant Cell.

[CR4] Cabada Gomez DA, Chavez MI, Cobos AN, Gross RJ, Yescas JA, Balogh MA, Indriolo E (2020). COPI complex isoforms are required for the early acceptance of compatible pollen grains in *Arabidopsis thaliana*. Plant Reprod.

[CR5] Chung KP, Zeng Y, Jiang L (2016). COPII paralogs in plants: functional redundancy or diversity?. Trends Plant Sci.

[CR6] Conger R, Chen Y, Fornaciari S, Faso C, Held MA, Renna L, Brandizzi F (2011). Evidence for the involvement of the Arabidopsis SEC24A in male transmission. J Exp Bot.

[CR7] Donohoe BS, Kang BH, Staehelin LA (2007). Identification and characterization of COPIa- and COPIb-type vesicle classes associated with plant and algal Golgi. Proc Natl Acad Sci USA.

[CR8] El-Kasmi F, Pacher T, Strompen G, Stierhof YD, Müller LM, Koncz C, Mayer U, Jürgens G (2011). Arabidopsis SNARE protein SEC22 is essential for gametophyte development and maintenance of Golgi-stack integrity. Plant J.

[CR9] Gao C, Cai Y, Wang Y, Kang BH, Aniento F, Robinson DG, Jiang L (2014). Retention mechanisms for ER and Golgi membrane proteins. Trends Plant Sci.

[CR10] Gimeno-Ferrer F, Pastor-Cantizano N, Bernat-Silvestre C, Selvi-Martínez P, Vera-Sirera F, Gao C, Perez-Amador MA, Jiang L, Aniento F, Marcote MJ (2017). α2-COP is involved in early secretory traffic in Arabidopsis and is required for plant growth. J Exp Bot.

[CR11] Jain Goyal M, Zhao X, Bozhinova M, Andrade-López K, de Heus C, Schulze-Dramac S, Müller-McNicoll M, Klumperman J, Béthune J (2020) A paralog-specific role of COPI vesicles in the neuronal differentiation of mouse pluripotent cells. Life Sci Alliance 3(9):e202000714. 10.26508/lsa.202000714 10.26508/lsa.202000714 PMC7368096 32665377

[CR12] Kitazawa D, Yamaguchi M, Mori H, Inoue YH (2012). COPI-mediated membrane trafficking is required for cytokinesis in Drosophila male meiotic divisions. J Cell Sci.

[CR13] Liu X, Tong M, Zhang A, Liu M, Zhao B, Liu Z, Li Z, Zhu X, Guo Y, Li R (2021). COPII genes *SEC31A/B* are essential for gametogenesis and interchangeable in pollen development in Arabidopsis. Plant J.

[CR14] Liu F, Li JP, Li LS, Liu Q, Li SW, Song ML, Li S, Zhang Y (2021a) The canonical α-SNAP is essential for gametophytic development in Arabidopsis. PLoS Genet 17(4):e1009505. 10.1371/journal.pgen.1009505 10.1371/journal.pgen.1009505 PMC8096068 33886546

[CR15] Moreno RD, Ramalho-Santos J, Sutovsky P, Chan EK, Schatten G (2000). Vesicular traffic and golgi apparatus dynamics during mammalian spermatogenesis: implications for acrosome architecture. Biol Reprod.

[CR16] Pereira C, Di Sansebastiano GP (2021). Mechanisms of membrane traffic in plant cells. Plant Physiol Biochem.

[CR17] Popoff V, Adolf F, Brügger B, Wieland F (2011) COPI budding within the Golgi stack. Cold Spring Harb Perspect Biol 3(11):a005231. 10.1101/cshperspect.a005231 10.1101/cshperspect.a005231 PMC3220356 21844168

[CR18] Rojek J, Tucker MR, Pinto SC, Rychłowski M, Lichocka M, Soukupova H, Nowakowska J, Bohdanowicz J, Surmacz G, Gutkowska M (2021). Rab-dependent vesicular traffic affects female gametophyte development in Arabidopsis. J Exp Bot.

[CR19] Sánchez-Simarro J, Bernat-Silvestre C, Gimeno-Ferrer F, Selvi-Martínez P, Montero-Pau J, Aniento F, Marcote MJ (2020). Loss of Arabidopsis β-COP function affects Golgi structure, plant growth and tolerance to salt stress. Front Plant Sci.

[CR20] Sánchez-Simarro J, Selvi P, Bernat-Silvestre C, Minguet EG, Aniento F, Marcote MJ (2022). Differential involvement of Arabidopsis β'-COP isoforms in plant development. Cells.

[CR21] Tanaka Y, Nishimura K, Kawamukai M, Oshima A, Nakagawa T (2013). Redundant function of two Arabidopsis COPII components, AtSec24B and AtSec24C, is essential for male and female gametogenesis. Planta.

[CR22] Woo CH, Gao C, Yu P, Tu L, Meng Z, Banfield DK, Yao X, Jiang L (2015). Conserved function of the lysine-based KXD/E motif in Golgi retention for endomembrane proteins among different organisms. Mol Biol Cell.

[CR23] Zhou Y, Fang W, Pang Z, Chen LY, Cai H, Ain NU, Chang MC, Ming R (2022) *AP1G2* affects mitotic cycles of female and male gametophytes in Arabidopsis. Front Plant Sci 13:924417. 10.3389/fpls.2022.924417 10.3389/fpls.2022.924417 PMC9301471 35873977

[CR24] Zimmermann P, Hirsch-Hoffmann M, Hennig L, Gruissem W (2004) GENEVESTIGATOR. Arabidopsis Microarray database and analysis toolbox. Plant Physiol 136: 2621. 10.1104/pp.104.046367 10.1104/pp.104.046367 PMC523327 15375207

